# Higher Intensity of Salt Stress Accompanied by Heat Inhibits Stomatal Conductance and Induces ROS Accumulation in Tomato Plants

**DOI:** 10.3390/antiox13040448

**Published:** 2024-04-10

**Authors:** Yankai Li, Fangling Jiang, Zhenxiang He, Yi Liu, Zheng Chen, Carl-Otto Ottosen, Ron Mittler, Zhen Wu, Rong Zhou

**Affiliations:** 1 College of Horticulture, Nanjing Agricultural University, Nanjing 210095, China; 2023204044@stu.njau.edu.cn (Y.L.); jfl@njau.edu.cn (F.J.); 18066074534@163.com (Z.H.); 2023104069@stu.njau.edu.cn (Y.L.); 2019204027@njau.edu.cn (Z.C.); 2 Department of Food Science, Aarhus University, Agro Food Park 48, 8200 Aarhus, Denmark; coo@food.au.dk; 3 Division of Plant Science and Technology, College of Agriculture, Food and Natural Resources, Bond Life Sciences Center, University of Missouri, Columbia, MO 65211, USA; mittlerr@missouri.edu

**Keywords:** tomato, combined heat and salt, stress intensity, stress duration, gas exchange, reactive oxygen species

## Abstract

Under natural conditions, abiotic stresses that limit plant growth and development tend to occur simultaneously, rather than individually. Due to global warming and climate change, the frequency and intensity of heat and salt stresses are becoming more frequent. Our aim is to determine the response mechanisms of tomato to different intensities of combined heat and salt stresses. The physiological and morphological responses and photosynthesis/reactive oxygen species (ROS)-related genes of tomato plants were compared under a control, heat stress, salt stress (50/100/200/400 mM NaCl), and a combination of salt and heat stresses. The stomatal conductance (g_s_) of tomato leaves significantly increased at a heat + 50 mM NaCl treatment on day 4, but significantly decreased at heat + 100/200/400 mM NaCl treatments, compared with the control on days 4 and 8. The O_2_^·−^ production rate of tomato plants was significantly higher at heat + 100/200/400 mM NaCl than the control, which showed no significant difference between heat + 50 mM NaCl treatment and the control on days 4 and 8. Ascorbate peroxidase 2 was significantly upregulated by heat + 100/200/400 mM NaCl treatment as compared with heat + 50 mM NaCl treatment on days 4 and 8. This study demonstrated that the dominant effect ratio of combined heat and salt stress on tomato plants can shift from heat to salt, when the intensity of salt stress increased from 50 mM to 100 mM or above. This study provides important information for tomato tolerance improvement at combined heat and salt stresses.

## 1. Introduction

As global climatic changes progress, the timing and intensity of heat stresses increase and become more unpredictable, severely affecting the growth, development, and yield of plants [1,2]. The effects of heat stress on plants are multifaceted and lead to changes in cellular structure and physiological function, such as protein denaturation, membrane integrity loss, and reduced cellular function [2,3,4,5]. The negative effects of soil salinization are also exacerbated by global climatic changes (e.g., heat waves), as well as rising sea levels, decreased water quality, and improper irrigation practices, affecting agricultural production in many areas of the world [6,7]. The effects of salt stress on plants include three main components: osmotic stress, ionic stress, and nutrient imbalances [8]. Salt stress can also interfere with plant metabolism by inducing stomatal closure to avoid ion uptake by roots and consequently suppress photosynthesis [1,7].

Under natural conditions, several stress factors that inhibit plant growth often simultaneously occur [9,10,11]. The interaction between two stress conditions can lead to either negative or positive effects on plant growth and productivity [12]. Thus, the mechanism of plants’ responses to combined stress cannot be inferred from a simple superposition of single stresses [13]. During agricultural production, large areas of lands affected by salinization are simultaneously subjected to heat stress [1,14]. There has been a great amount of research on responsive mechanisms of plants to single heat or salt stresses [2,3,6,8]. However, few studies have focused on plant responses to combined heat and salt stresses [13,14,15,16]. Rivero er al. (2014) illustrated that combined heat and salt stresses significantly increased the accumulation of osmoprotectants, such as alginate, resulting in a significant increase in antioxidant capacity of tomatoes [14], and Jan et al. (2021) indicated that significant accumulation of flavonoid metabolites in rice plants occurred due to overexpression of the *F3H* gene, which alleviated the negative effects due to combined heat and salt stresses [15]. Suzuki et al. (2016) illustrated that transcripts specifically accumulated under combined heat and salt stresses were associated with the phytohormone abscisic acid in Arabidopsis [16]. Pardo et al. (2024) identified a number of important ABA-independent transcription factors (TFs) in the specific response of tomato to combined heat and salt stresses, including TFs SlMYB50 and SlMYB86, which are directly involved in the flavonol biosynthetic pathway [17]. Moreover, our recent study showed that oxidative phosphorylation, an important pathway regulating respiratory processes, played a key role in the response of tomatoes to combined salt and heat stress [13]. However, to date, most studies of stress combination, including heat and salt, focused on a single level of each of the individual stresses, which prevented a clear evaluation of the relative dominance between each of the stresses involved in the stress combination.

The negative effects of combined stresses on plants can be dominated by one stressor [1,16]. For example, stomata of tomato were kept open during heat stress (32 °C/26 °C, day/night), which reduced leaf temperature by increasing transpiration rate [18]. In contrast, drought stress led to the closure of stomata, which reduce leaf transpiration rate to maintain water content of the leaves [18,19]. Under the combined drought and heat stress, however, the stomata of tomato closed, and leaf photosynthesis decreased to almost zero [18]. This finding suggested that, at least with respect to stomatal responses, the drought stress dominated when it was combined with heat stress (32 °C/26 °C) impacting photosynthetic activity [18]. By comparison, the stomatal conductance and photosynthetic capacity of tomatoes were significantly reduced at combined heat (32 °C/26 °C) and salt (200 mM NaCl) stress for 8 days, corresponding to the responses of plants at single salt stress instead of single heat stress, thereby suggesting salt stress as the dominant stressor [13]. By contrast, transcriptomic and metabolomic responses of tomatoes to a combined heat (36 °C) and salt (200 mM NaCl) stresses were more similar to those under heat stress (36 °C) for 48 h, suggesting that when it comes to transcriptional/metabolomics responses, heat stress was the dominant stressor [13]. The predominant role of each different stressor could therefore be shifted from heat to salt stress when the two stresses are combined during prolonged stress combination episodes; however, the underlying regulatory mechanisms controlling these processes remain unclear. How increasing salt stress intensity alters this balance of dominance requires, therefore, further investigation.

The plant photosynthetic machinery is very sensitive to stressful environments, mainly in terms of photochemical reactions and carbon cycling processes [20]. ROS play an indispensable role as signaling molecules in the regulation of many biological processes such as growth, development, and response to biotic/abiotic stresses in plant [4]. ROS-induced damage was extensive, targeting all biomolecules such as lipids, proteins, and DNA, disrupting cellular integrity and ultimately leading to cell death [4]. Heat stress results in inactivation of photosystem II (PSII) electron acceptors and donors, as well as disabling carbon cycling enzymes and decreases ribulose-1,5-bisphosphate carboxylase/oxygenase (Rubisco) activity, which is accompanied by increased ROS levels and oxidative damage [20]. The manganese-stabilizing protein (PsbO) is believed to increase the efficiency of oxygen evolution and to stabilize the PSII catalytic active site during stress [21], and isozymes of ascorbate peroxidase (APX), which is a central component of the antioxidative system, play an important role in ROS scavenging in the various plant subcellular compartments [22]. This study investigated and compared the responses of tomatoes under a control, heat stress, four intensities of salt stress, and their combination, from the perspective of photosynthesis, ROS, expression of key genes (e.g., *PsbO*, *APX1*, *rbcS*), stomatal regulation, and plant morphology. Our goal is to unravel the response mechanisms of tomatoes to different intensities of combined heat and salt stresses, with the following working hypotheses: (1) plants exhibit unique responses under different intensities of combined heat and salt stress; and (2) the dominant stressor of the combined heat and salt stresses can be altered with the change in salt stress intensity. Our research aims to contribute to a better understanding of how plants respond to different intensities of environmental stresses during stress combination and accelerate the breeding process of tomato varieties with combined stress tolerance.

## 2. Materials and Methods

### 2.1. Plant Materials

Experiments were conducted in the greenhouse of Nanjing Agricultural University. Soil media were prepared in the ratio of charcoal: vermiculite: perlite (*V*/*V*/*V*) = 2:1:1. Tomato seeds of ‘QianXi’ (CL032, Known-You, Xiamen, China) were sown in 72-hole trays. The seedlings with true leaves were irrigated with nutrient solution every five days. The nutrient solution referred to the Japanese garden formula (945 mg/L Ca(NO_3_)_2_•4H_2_O, 809 mg/L KNO_3_, 153 mg/L NH_4_H_2_PO_4_, 493 mg/L MgSO_4_•7H_2_O, 13.9 mg/L FeSO_4_•7H_2_O, 18.6 mg/L Na_2_-EDTA, 2.86 mg/L H_3_BO_3_, 2.13 mg/L MnSO_4_•4H_2_O, 0.22 mg/L ZnSO_4_•7H_2_O, 0.08 mg/L CuSO_4_•5H_2_O, 0.02 mg/L (NH_4_)_6_Mo_7_O_24_•4H_2_O). On 23 days after sowing, the seedlings with three true leaves were transplanted into nutrient pots (6.5 cm long × 6.5 cm wide × 8 cm high) using the same media and acclimated for 4 days.

### 2.2. Experimental Design

In total, 240 seedlings with uniform size were divided into 10 groups. Half of the plants (five groups) continuously grew in the same environment as above, with 0, 50, 100, 200, and 400 mM NaCl solution applied for each group, corresponding to C, S1, S2, S3, and S4 treatment, respectively. The remaining plants (five groups) were covered by square shed (2.6 m long × 0.5 m wide × 0.4 cm high) using transparent PE film and steel frames to simulate higher growth temperature. Each group was applied with 0, 50, 100, 200, and 400 mM NaCl solution, respectively, corresponding to H, HS1, HS2, HS3, and HS4. Plants under the salt and combined stress treatment were irrigated with 100 mL NaCl solution at 9:00 a.m. on days 1, 3, 5, and 7. The control and heat stress treatment groups were treated with equal amounts of double distilled water (ddH_2_O). Real-time temperatures were monitored using the TH20BL-EX-P67 model thermohygrometer (TH20BL, Miaoxin, Wenzhou, China) and shown in Figure S1. Light intensity for all plant groups was 300–350 μmol m^−2^ s^−1^ during the daytime and relative humidity in the greenhouse was between 55–75%.

### 2.3. Measurements

At the middle stage (day 4) and in the late stage (day 8) of stresses, gas exchange parameters, ROS accumulation, and morphological traits were measured, and leaf samples were collected for qRT-PCR. All data were collected from the apical leaflet of the second fully expanded leaf counting from the top.

#### 2.3.1. Leaf Photosynthetic Activity

Net photosynthetic rate (*P_n_*), stomatal conductance (*g_s_*), intercellular CO_2_ concentration (*C_i_*), and transpiration rate (*E*) of tomato leaves were measured using a portable gas exchange analyzer (Li-6400XT, LiCor, Lincoln, NE, USA) on the morning of day 4 and day 8 after the treatments. The leaf chamber CO_2_ concentration was 400 ppm with 1000 μmol m^−2^ s^−1^ light intensity, and the flow rate was 500 μmol s^−1^. The leaf chamber temperature was set to 25 °C when measuring the control and salt stress groups, which was set to 35 °C for the heat and combined stress groups.

#### 2.3.2. Leaf H_2_O_2_ Content and O_2_^·−^ Production Rate

Leaf H_2_O_2_ content was determined according to the method of Chakrabarty et al. (2008) [23]. In detail, 0.2 g of leaves were ground in liquid nitrogen and 2 mL pre-cooled 0.1% trichloroacetic acid (TCA) was added. Then, the samples were centrifuged at 12,000 rpm (revolutions per minute) for 15 min. Then, 0.5 mL supernatant were taken and added with 2 mL 1 M KI solution and 0.5 mL 100 mM phosphate buffer. After the reaction in darkness for 1 h, the absorbance values at 390 nm were determined using microporous plate detecting instrument (Cytation3, Bio Tek, Winooski, VT, USA) with 0.1% TCA as reference. A standard curve of H_2_O_2_ concentration–absorbance value (x-y) was made, and the leaf H_2_O_2_ content was calculated using the curve function.

The O_2_^·−^ production rate was determined according to Ke et al. (2007) [24]. A 0.2 g fresh leaves were placed in a mortar, and 1 mL 50 mM pre-cooled phosphate buffer (pH 7.8) was added. The homogenate was centrifuged at 4 °C and 12,000 rpm for 20 min to obtain the extracts. The 0.5 mL extract was taken, which was mixed with 0.5 mL phosphate buffer (50 mM, pH 7.8) and 1 mL 10 mM hydroxylamine hydrochloride solution. The reaction solution was mixed and shaken well, and then incubated at 25 °C for 1 h. Then, 2 mL ether was added, followed by 1 mL 17 mM sulfanilic acid, 1 mL 7 mM α-naphthylamine. Finally, the reaction solution was incubated at 25 °C for 20 min and then centrifuged at 3000 rpm for 3 min. The absorbance value at 530 nm was determined by taking the pink aqueous solution.

#### 2.3.3. Total RNA Extraction, Reverse Transcription, and qRT-PCR

To measure changes in steady-state transcript levels in response to the different stress intensities and durations, we selected key genes in the photosynthetic, ROS, and Rubisco pathways in response to the combined heat and salt stresses from the previous sequencing data (NCBI accession number: PRJNA937899, https://dataview.ncbi.nlm.nih.gov/object/PRJNA937899, Created: 23 February 2023) for qRT-PCR [13], and the key genes information were shown in Table S1. Tomato leaf RNA was extracted using RL lysate (AF504A, Proteinssci, Shanghai, China) and reverse transcribed into cDNA using the HiScript Ⅱ Reverse Transcriptase First Strand cDNA Synthesis Kit (Vazyme, Nanjing, China). Reactions were performed in a reverse transcription system (Eppendorf, Hamburg, Germany). Primer 3 (Primer-E Ltd., Plymouth, UK) was applied for primer design, and the primer sequences were shown in Table S2.

The qRT-PCR was performed using the TOROGreen qPCR Master Mix kit (Toroivd, Shanghai, China). Reactions were performed in a QuantStudio^®^ 3 real-time PCR system (Thermo Fisher Scientific, Singapore). The 10 µL Reaction system included 5 µL TOROGreen qPCR Master Mix, 1 µL cDNA (at 10-fold dilution), 0.4 µL upstream and downstream primers, and 3.2 µL ddH_2_O. The reaction procedure was as follows: 95 °C pre-denaturation for 60 s, followed by 40 cycles of 10 s at 95 °C and 30 s at 60 °C. A melting curve analysis was performed using the default parameters (5 s at 95 °C and 1 min at 65 °C) in the same way as Li et al. (2023) [13].

#### 2.3.4. Morphological Characterization of Plants

Plant height was measured using a ruler (accuracy 0.1 cm) on days 4 and 8. The stem diameter was determined using vernier calipers (accuracy 0.1 mm). Tomato plants were cut at the cotyledon node and the above-ground fresh weight was determined using an electronic balance. The above-ground samples were placed in an oven at 105 °C for 15 min and then dried at 65 °C until the plant mass was constant. Then, the samples were weighed as above-ground dry weight.

### 2.4. Data Analysis

Data were analyzed and graphed using Origin (Origin 2022, Northampton, MA, USA). The analysis of variance (ANOVA) was applied using IBM SPSS Statistics 16.0 (SPSS Inc., Chicago, IL, USA) according to Duncan’s test with a significance threshold of *p* < 0.05. The relative expression of key genes was calculated using the 2^−∆∆Ct^ method. All data contained three biological replications, where the measurements of leaf photosynthetic parameters contained a further three technical replications.

## 3. Results

### 3.1. Changes in Photosynthetic Activity in Tomato Plants Subjected to Stress Combination

**Figure 1:**
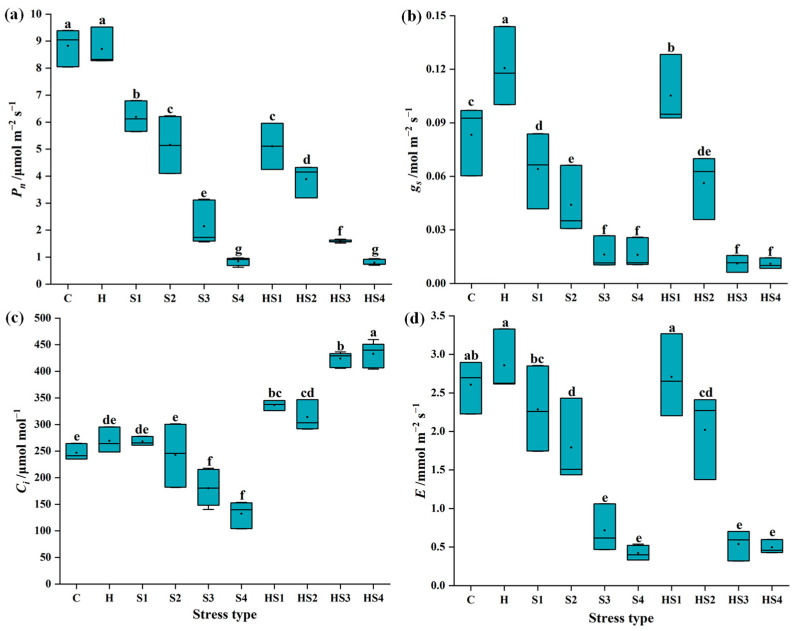
(**a**) Net photosynthetic rate (*P_n_*), (**b**) stomatal conductance (*g_s_*), (**c**) intercellular CO_2_ concentration (*C_i_*), and (**d**) transpiration rate (*E*) of tomato plants at 4 days post stress/stress combination initiation. C and H indicate the control and heat stress, respectively. S1/S2/S3/S4 indicate 50/100/200/400 mM NaCl treatments, respectively. HS1/HS2/HS3/HS4 indicated the combination of heat stress with four corresponding intensities of salt stress, respectively. Dots and lines in the boxes show the mean and median, respectively (*n* = 9). Different lowercase letters indicated differences at the *p* < 0.05 level according to Duncan’s test.

The net photosynthetic rate (*P_n_*) of tomato leaves was significantly lower at four intensities of salt stress and combined stress compared to the control and heat stress on day 4 (Figure 1a). The stomatal conductance (*g_s_*) of tomato leaves was significantly higher at heat stress but significantly lower at all four intensities of salt stress, compared with the control on day 4 (Figure 1b). The *g_s_* was significantly higher in tomato at heat + 50 mM NaCl treatment, but significantly lower at the other three intensities of combined stress, compared with the control on day 4 (Figure 1b). The intercellular CO_2_ concentration (*C_i_*) of tomato leaves was significantly reduced at 200 mM and 400 mM NaCl, compared with the control on day 4 (Figure 1c). The transpiration rate (*E*) of tomato leaves significantly decreased at 100/200/400 mM NaCl treatments as well as heat + 100/200/400 mM NaCl treatments compared to the control on day 4 but maintained at control levels at heat + 50 mM NaCl (Figure 1d).

**Figure 2:**
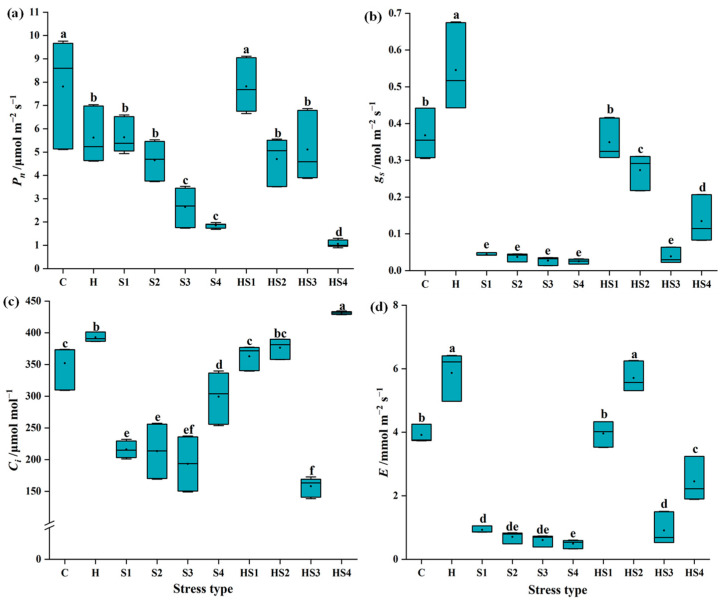
(**a**) Net photosynthetic rate (*P_n_*), (**b**) stomatal conductance (*g_s_*), (**c**) intercellular CO_2_ concentration (*C_i_*), and (**d**) transpiration rate € of tomato plants at 8 days post stress/stress combination initiation. C and H indicate the control and heat stress, respectively. S1/S2/S3/S4 indicate 50/100/200/400 mM NaCl treatments, respectively. HS1/HS2/HS3/HS4 indicated the combination of heat stress with four corresponding intensities of salt stress, respectively. Dots and lines in the boxes show the mean and median, respectively (*n* = 9). Different lowercase letters indicated differences at the *p* < 0.05 level according to Duncan’s test.

The *P_n_* of tomato leaves was significantly lower at all stress treatments (except heat + 50 mM NaCl treatment) than the control on day 8 (Figure 2a). The *g_s_* of tomato leaves was significantly higher at heat stress than the control on day 8 (Figure 2b), but *g_s_* was significantly lower at all intensities of salt stress than the control on day 8 (Figure 2b). The *g_s_* of leaves was significantly lower at the combined stress (except heat + 50 mM NaCl treatment) than the control and heat stress on day 8 (Figure 2b). The *C_i_* of tomato leaves significantly reduced at all the four intensities of individual salt stress as compared with the control on day 8 (Figure 2c). The *C_i_* of tomato leaves significantly reduced at heat + 200 mM NaCl treatment as compared with the control on day 8 (Figure 2c). The *E* of tomato leaves was significantly higher at individual heat and heat + 100 mM NaCl treatments, but significantly lower at the four intensities of individual salt stress and heat + 200/400 mM NaCl treatments, as compared to the control on day 8 (Figure 2d).

### 3.2. Changes of H_2_O_2_ and O_2_^·−^ Levels of Tomato Plants Subjected to Stress Combination

**Figure 3:**
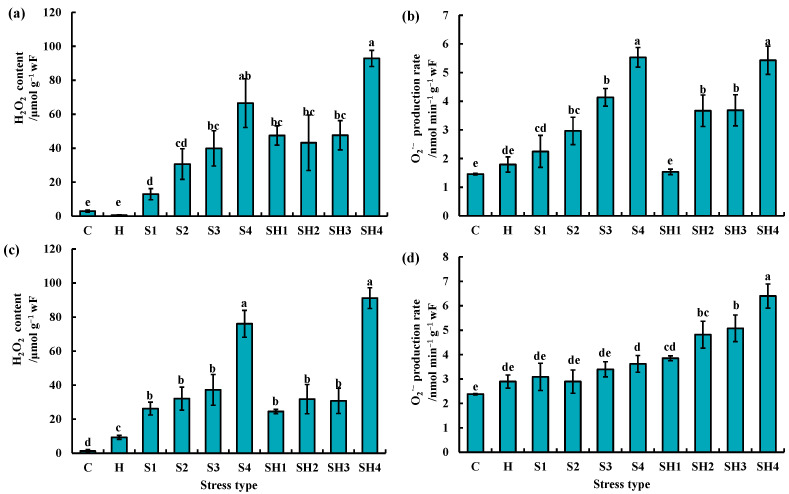
(**a**,**c**) H_2_O_2_ content and (**b**,**d**) O_2_^·−^ levels of tomato leaves at 4 and 8 days post stress/stress combination initiation. The 4 d of stress: (**a**,**b**); the 8 d of stress: (**c**,**d**). The data were mean ± standard error (*n* = 3). C and H indicate control and heat stress, respectively. S1/S2/S3/S4 indicate 50/100/200/400 mM NaCl treatments, respectively. HS1/HS2/HS3/HS4 indicated the combination of heat stress with four corresponding intensities of salt stress, respectively. Different lowercase letters indicated differences at the *p* < 0.05 level according to Duncan’s test.

The H_2_O_2_ content of tomato leaves was significantly higher at all the four intensities of salt stress and combined stress than the control and heat stress on day 4 (Figure 3a). The O_2_^·−^ levels in tomato leaves were significantly higher at all the four intensities of salt stress and combined stress (except heat + 50 mM NaCl treatment) than the control on day 4 (Figure 3b). The O_2_^·−^ levels in tomato leaves significantly reduced at heat + 50 mM NaCl treatment, as compared with 50 mM NaCl treatment on day 4 (Figure 3b).

The H_2_O_2_ content of tomato leaves was significantly higher at all the stresses than the control on day 8 (Figure 3c). The O_2_^·−^ levels in tomato leaves were significantly higher at 400 mM NaCl treatment and all intensities of combined stress than the control on day 8 (Figure 3d). The O_2_^·−^ levels in tomato leaves did not significantly differ at the heat + 50 mM NaCl treatment, as compared with the 50 mM NaCl treatment on day 8 (Figure 3d). The O_2_^·−^ levels in tomato leaves were significantly higher at heat + 100 mM NaCl treatment, as compared with 100 mM NaCl treatment on day 8 (Figure 3d).

### 3.3. Changes in the Steady-State Levels of Transcripts Encoding Different Photosynthesis/ROS-Related Proteins

**Figure 4:**
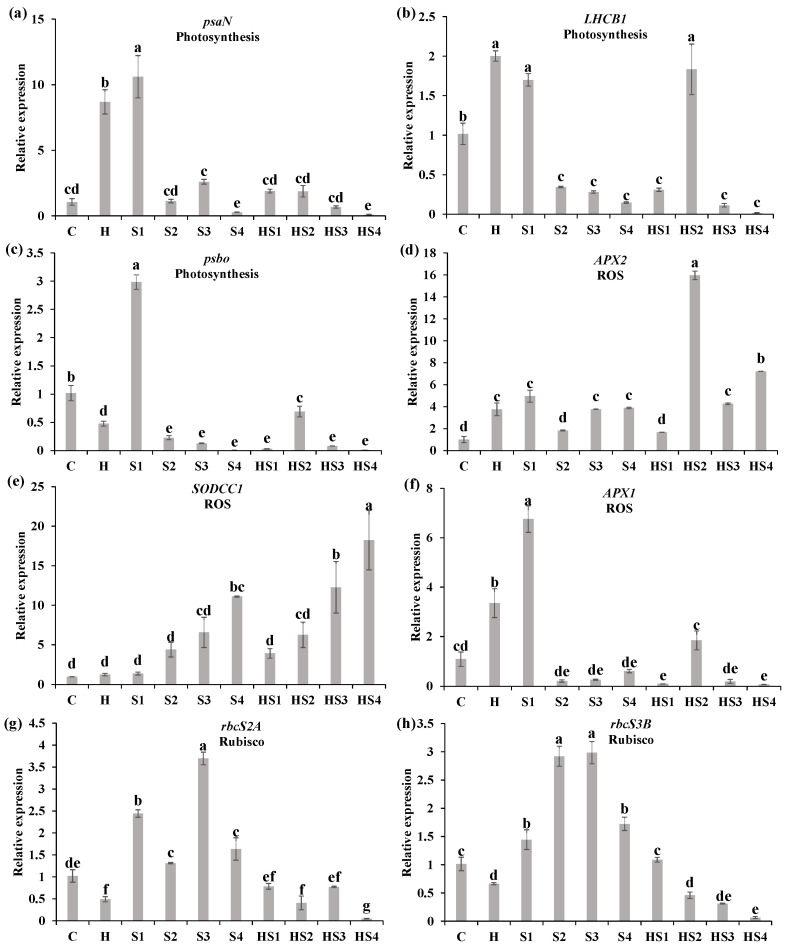
Relative expression level of (**a**–**c**) photosynthesis, (**d**–**f**) ROS, and (**g**,**h**) Rubisco pathway-related genes of tomato plants at the ten treatments for 4 days using qRT-PCR. The functional pathway of the gene was labeled below the gene symbol. C and H indicate the control and heat stress, respectively. S1/S2/S3/S4 indicate 50/100/200/400 mM NaCl treatments, respectively. HS1/HS2/HS3/HS4 indicated the combination of heat stress with four corresponding intensities of salt stress, respectively. The data were mean ± standard error (*n* = 3). Different lowercase letters indicated differences at the *p* < 0.05 level according to Duncan’s test.

The relative expression of *psaN* (photosystem I reaction centre subunit N) was significantly lower at 400 mM NaCl treatment and heat + 400 mM NaCl treatment than the control on day 4 (Figure 4a). The relative expression of *LHCB1* (chlorophyll a-b binding protein) was significantly lower at heat + 50 mM NaCl and heat + 200/400 mM NaCl treatments, which was significantly higher at heat + 100 mM NaCl treatment, compared with the control on day 4 (Figure 4b). The relative expression of *PsbO* (33 kDa precursor protein of oxygen-evolving complex) significantly decreased at all the stress types (except 50 mM NaCl treatment) compared with the control on day 4 (Figure 4c). The relative expression of *APX2* (L-ascorbate peroxidase 2, cytosolic) was up-regulated by heat + 100/200/400 mM NaCl treatments as compared with the control on day 4 (Figure 4d). The relative expression of *SODCC1* (Cu^2+^/Zn^2+^ Superoxide dismutase 1, cytosolic, chloroplastic) was significantly higher at 400 mM NaCl treatment, heat + 200/400 mM NaCl treatments than the control on day 4 (Figure 4e). The relative expression of *APX1* (cytosolic ascorbate peroxidase 1) significantly reduced at heat + 50 mM/400 NaCl treatments as compared with the control on day 4 (Figure 4f). The relative expression of *rbcS2A* (ribulose bisphosphate carboxylase small chain 2A) and *rbcS3B* (ribulose bisphosphate carboxylase small chain 3B) was up-regulated by single salt stress but down-regulated under combined stress (HS2 and HS4), compared with the control (Figure 4g,h).

**Figure 5:**
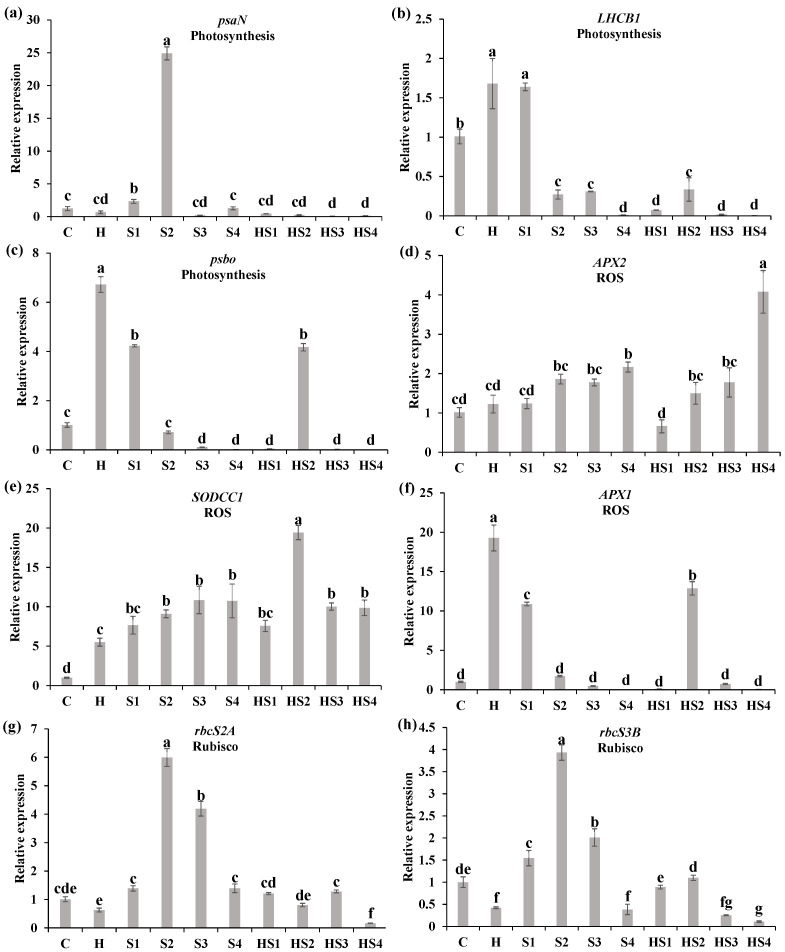
Relative expression level analysis of (**a**–**c**) photosynthesis, (**d**–**f**) ROS, and (**g**,**h**) Rubisco pathway-related genes of tomato plants at the ten treatments for 8 days using qRT-PCR. The functional pathway of the gene was labeled below the gene symbol. C and H indicate the control and heat stress, respectively. S1/S2/S3/S4 indicate 50/100/200/400 mM NaCl treatments, respectively. HS1/HS2/HS3/HS4 indicated the combination of heat stress with four corresponding intensities of salt stress, respectively. The data were mean ± standard error (*n* = 3). Different lowercase letters indicated differences at the *p* < 0.05 level according to Duncan’s test.

The relative expression of *psaN* and *PsbO* was significantly lower at heat + 200/400 mM NaCl treatments than the control on day 8 (Figure 5a,c). The *LHCB1* was significantly downregulated by all intensities of individual salt stress (except for the 50 mM NaCl) and combined stress as compared with the control on day 8 (Figure 5b). The relative expression of *APX2* and *SODCC1* was significantly higher at both 400 mM NaCl treatment and heat + 400 mM NaCl treatment than the control on day 8 (Figure 5d,e). The relative expression of *APX1* was significantly higher at heat stress, 50 mM NaCl, and heat + 100 mM NaCl treatments than the control on day 8 (Figure 5f). The relative expression of *rbcS2A* and *rbcS3B* was up-regulated by single salt stress (mainly S2/S3) but down-regulated under combined stress (mainly HS4), compared with the control (Figure 5g,h).

### 3.4. Morphological Changes in Tomato Plants Subjected to Stress Combination

**Figure 6:**
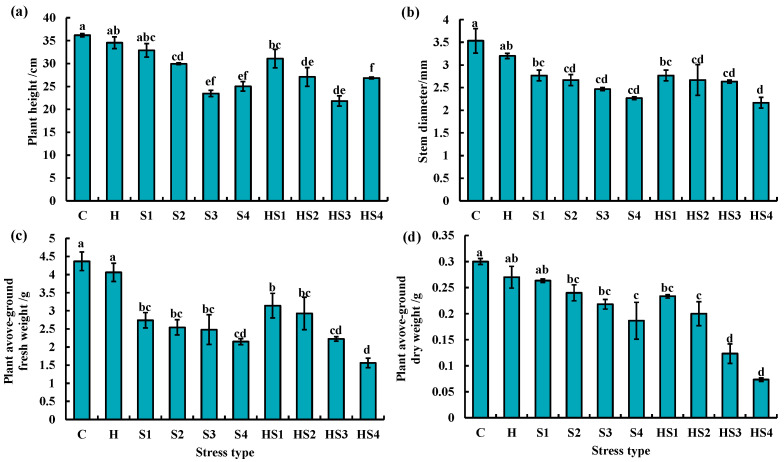
(**a**) Plant height, (**b**) stem diameter, (**c**) above-ground fresh weight and (**d**) above-ground dry weight of tomato plants at the ten treatments for 4 days. The data were mean ± standard error (*n* = 3). C and H indicate the control and heat stress, respectively. S1/S2/S3/S4 indicate 50/100/200/400 mM NaCl treatments, respectively. HS1/HS2/HS3/HS4 indicated the combination of heat stress with four corresponding intensities of salt stress, respectively. Different lowercase letters indicated differences at the *p* < 0.05 level according to Duncan’s test.

On day 4, plant height was significantly lower at 100/200/400 mM NaCl solution treatments and all the intensities of combined stress than the control (Figure 6a). Stem thickness and above-ground fresh weight was significantly lower in tomato at all the four intensities of individual salt and combined stress than the control and heat stress on day 4 (Figure 6b,c). Tomato aboveground dry weight was significantly reduced by the 100/200/400 mM NaCl treatments and all intensities of combined stress than the control on day 4 (Figure 6d).

**Figure 7:**
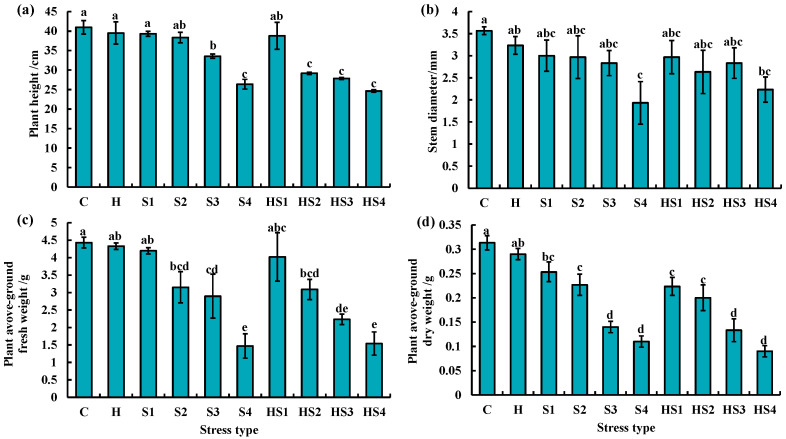
(**a**) Plant height, (**b**) stem diameter, (**c**) above-ground fresh weight and (**d**) above-ground dry weight of tomato plants at the ten treatments for 8 days. The data were mean ± standard error (*n* = 3). C and H indicate the control and heat stress, respectively. S1/S2/S3/S4 indicate 50/100/200/400 mM NaCl treatments, respectively. HS1/HS2/HS3/HS4 indicated the combination of heat stress with four corresponding intensities of salt stress, respectively. Different lowercase letters indicated differences at the *p* < 0.05 level according to Duncan’s test.

Tomato plant height was significantly lower at 200/400 mM NaCl treatments and combined stress (except heat + 50 mM NaCl treatment) than the control on day 8 (Figure 7a). Stem thickness of tomato was significantly lower at 400 mM NaCl and heat + 400 mM NaCl treatments than the control on day 8 (Figure 7b). The aboveground fresh weight of tomato was significantly lower in tomato at 200 mM, 400 mM NaCl, heat + 200/400 mM NaCl treatments as compared with the control and heat stress on day 8 (Figure 7c). All the four intensities of salt stress (except 50 mM NaCl treatment) and combined stress significantly inhibited the aboveground dry weight of tomato plants as compared with the control and heat stress on day 8 (Figure 7d).

## 4. Discussion

With global warming and climate change, unfavorable environmental conditions are occurring more frequently in different agroecosystems [1]. Heat and soil salinization are two major environmental factors contributing to agricultural losses globally [3,25]. Despite numerous and in-depth studies on the physiological and genetic response mechanisms of plants subjected to single heat or salt stresses, little is known about the effects of combined heat and salt stresses on plants, especially under different stress intensities. Therefore, this study was carried out to investigate the morphological, physiological and transcript expression responses of tomato plants to heat stress, four intensities of salt stresses and four intensities of salt stress combined heat and salt stresses.

### 4.1. Physiological and Molecular Responses of Tomato to Combined Heat and Salt Stress Are Affected by Stress Intensity and Duration

The leaf photosynthetic capacity of plants is a valuable indicator of the stress effects, as the photosynthetic system is closely linked to changes in biomass [14]. Restrictions to any part of the photosynthetic processes by stress can limit plant growth [1,18]. The mechanisms by which different types of stress impact the plant photosynthetic system differ [26]. On one hand, salt stress mainly causes osmotic and ionic stress to plants, which induces stomatal closure and thereby limits ion uptake and photosynthetic rates [8,20]. On the other hand, heat stress mainly affects the biochemical reactions of leaf photosynthesis [27]. Depending on the intensity and duration of stress, heat stress can irreversibly cause structural damage and reduce activities of PSII and Rubisco etc. [27,28]. Intercellular CO_2_ concentration (*Ci*) of tomato leaves was significantly reduced under single salt stress (mainly S3/S4) but significantly higher under combined stress, compared with the control (Figure 1 and Figure 2). This suggests that the temperature factor plays an important role in regulating *Ci* of tomato leaves under combined stress. The net photosynthetic rate (*P_n_*) of tomatoes was significantly lower at heat + 50/100/200 mM NaCl than the individual NaCl treatments on day 4 (Figure 1a). However, when the salt stress intensity increased to 400 mM NaCl, there was no significant difference in tomato *P_n_* between the individual salt and combined stress (Figure 1a). This finding indicates that response mechanisms of plants to combined heat and salt stress are distinct and potentially regulated by the salt stress intensity, leading to difference in photosynthetic system responses and finally plant biomass accumulation.

**Figure 8:**
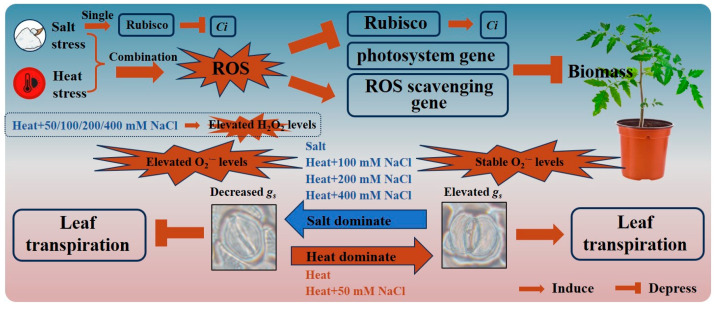
Effects of combined heat and salt stress on *Ci*, *g_s_*, ROS, the expression of key transcripts, and the biomass of tomato. Different arrow types indicated the induction or repression effects of stress.

The physiological and morphological responses of plants to abiotic stresses are primarily controlled by transcriptional activation or repression of genes, different signal transduction responses, and metabolic changes [29]. During this process, many metabolic mechanisms are coordinated, including rapid changes in gene expression, ion regulation, and protein activation and inactivation [13,26]. The steady-state levels of key transcripts belonging to different tomato photosynthetic pathways (*psaN*, *LHCB1*, *psbo*) were significantly down-regulated in tomato plants at heat + 50 mM NaCl treatment compared with 50 mM NaCl treatment on day 4 and 8 (Figure 4a–c and Figure 5a–c). Zandalinas et al. (2020) reviewed the transcriptional regulatory networks of plants altered by combined stresses, indicating that the simultaneous application of two different stress types to plants would lead to the exclusive activation of many transcripts [26]. The steady-state levels of *Rubisco* were not significantly different under single salt stress but significantly down-regulated under combined stress, compared with the control (Figure 4g,h and Figure 5g,h). This suggests that Rubisco effectiveness was reduced due to heat damage under combination stress, therefore the ability to assimilate external CO_2_ reduced, indirectly leading to increased *Ci* (Figure 8) [27]. We demonstrated that changes in transcript expression were affected not only by the type of combined stress, but also by the intensity and duration of each stressor.

We found that heat + 200 mM NaCl treatment significantly reduced the above-ground dry weight of tomato plants compared with 200 mM NaCl treatment on day 4 (Figure 6d). A similar trend was observed between 400 mM NaCl and heat + 400 mM NaCl treatment (Figure 6d). This finding suggested that the combined stresses produced more severe damage indicative by biomass accumulation of tomato plants at middle stage of stresses. In comparison, Lopez et al. (2021) found that heat (35 °C) + 75 mM NaCl treatment increased the plant biomass of tomato compared with single salt stress for 14 days [29]. The above two results revealed that the distinct intensity of salt stress could be a key factor in determining the effects of combined stress on tomato plants.

### 4.2. The Dominant Stressor of the Combined Heat and Salt Stress Can Change with Increasing Salt Stress Intensity

The effects of combined stresses on plants cannot be simply inferred from the effects of each of the single stresses composing the stress combination due to the different interactions between different stressors [30]. When exposed to a multifactorial combination of stresses, morphological, physiological, and transcriptional changes in plants tended to be unique, but plants could also behave more similarly to one of the single stressors [18,26]. We found a significant increase in stomatal conductance (*g_s_*) in tomato at heat stress and a significant decrease in *g_s_* at salt stress compared with the control (Figure 1b and Figure 2b), which was in agreement with previous studies [19,20]. When tomato plants were exposed to the combined heat and salt stress, the opening or closing states of stomata were affected by the intensity of salt stress (Figure 1b and Figure 2b). The stomatal conductance of tomato leaves significantly increased (i.e., stomata opened) at heat + 50 mM NaCl treatment, but significantly decreased (i.e., stomata closed) at heat + 100/200/400 mM NaCl treatment compared with the control on day 4 (Figure 1b). Thus, we conclude that heat is the dominant stress factor in regulating stomatal opening or closing of tomato at the heat + 50 mM NaCl treatment. However, when the salt stress intensity in the combined heat and salt stress increases (including 100/200/400 mM NaCl), salt becomes the dominant stressor (Figure 8). Increased *g_s_* indirectly promoted an increase in the rate of transpiration € in tomatoes, which promoted leaf cooling under heat stress [1,20]. The salt stress and heat + 100/200/400 NaCl treatments elevated ROS (mainly O_2_^·−^ levels) and decreased stomatal conductance in tomato leaves, which inhibited the transpiration rate and leaf cooling of the plant, thus potentially indirectly blocked the excess uptake of ions (Figure 8). Signals of ROS accumulation could have therefore altered the expression levels of photosystem-related genes and ROS scavenging system-related genes (Figure 4 and Figure 5), as well as stomatal responses, which ultimately affected the biomass of tomato plants (Figure 8).

ROS are crucial in abiotic and biotic stress sensing, which integrates different environmental signals and activates stress response networks, thus contributing to the establishment of defense mechanisms and plant resilience [4]. We found that there was no significant difference in the O_2_^·−^ production rate of tomato plants between the control/individual heat stress and heat + 50 mM NaCl treatments on day 4 (Figure 3b). When the salt stress intensity was 100 mM NaCl and above, however, the O_2_^·−^ production rate in tomato under combined stress was significantly higher than the control and individual heat stress (Figure 3b,d). In agreement, the expression of *APX2* and *SODCC1* was significantly upregulated by heat + 200/400 mM NaCl treatment compared with the control, but it was not significant different at heat + 50 mM NaCl treatment on day 4 (Figure 4d,e). This suggests that the increased intensity of salt in the combined stresses led to a significant accumulation of ROS and further activated the expression of ROS scavenging-related genes, as well as regulated leaf gas exchange. This finding suggests that ROS plays a crucial role in mediating the regulation of stomatal opening or closing by different intensities of combined stresses. Low O_2_^·−^ levels (e.g., at HS + 50 mM NaCl) could support stomatal opening, while high O_2_^·−^ and any H_2_O_2_ levels in this study support stomatal closing (Figure 8). Different ROS and other signals were generated in various cellular compartments under different stimuli, which can trigger stress-specific signal transduction pathways and activate stress-specific adaptation and defense mechanisms [31,32]. In summary, different types of ROS could mediate the regulation of stomatal opening or closing in tomato, depending on stress type, stress intensity, and duration, and this could be crucial for controlling plant responses to different intensities of salinity stress during the stress combination with heat.

## 5. Conclusions

The effects of combined heat and salt stress on tomato plants are not a simple superposition of each of the single stresses involved. Different intensities of salt stress combined with heat stress have differential effects on tomatoes, which altered the photosynthetic response of the tomato and ultimately led to reduced growth and biomass. Heat was the dominant stress factor in stomatal regulation of tomato at the combined heat and salt stress when 50 mM NaCl was applied. However, when salt stress intensity increased (100/200/400 mM NaCl), salt became the dominant stressor controlling stomatal responses during heat and salt stress combination. The stress type, stress intensity, duration, and type of ROS accumulated were therefore all possible factors controlling stomatal opening or closing in tomatoes.

## Data Availability

Data were contained within the article.

## Supplementary Materials

The following supporting information can be downloaded at: https://www.mdpi.com/article/10.3390/antiox13040448/s1, Figure S1: Environmental temperature during stress treatments. Red color represents temperatures of high heat and combined stress, blue color represents temperatures of the control and salt stress. Table S1: Information on key genes in photosynthesis, ROS, and Rubisco pathways in response to combined heat and salt stresses. Table S2: qRT-PCR primer sequence information.
