# Supplementary material for: Higher Intensity of Salt Stress Accompanied by Heat Inhibits Stomatal Conductance and Induces ROS Accumulation in Tomato Plants
Source: Antioxidants (Basel). 2024 Apr 10;13(4):448. doi: 10.3390/antiox13040448 (PMC11047744; doi:10.3390/antiox13040448)
Supplement: Supplementary file 1 [file antioxidants-13-00448-s001.zip › antioxidants-2909436-supplementary.pdf]

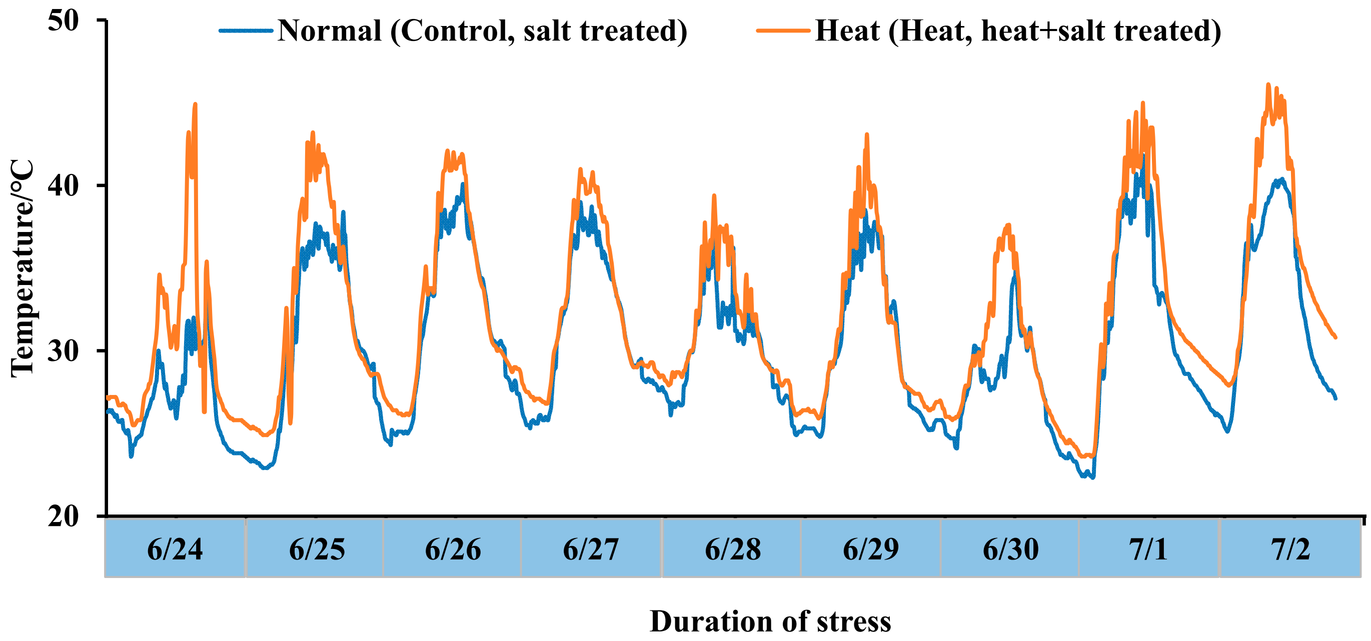

Figure S1. Environmental temperature during stress treatments. Blue colour represents temperature under control and salt stress. Orange colour represents the temperature under heat stress and the combined heat and salt stress. The x-axis shows the 8 d duration of the stress treatment from 24 June 2023 to 2 July 2023.

Table S1. Information on key genes in photosynthesis, ROS and Rubisco pathways in response to combined heat and salt stresses

Gene ID	Gene Name	Description
Solyc08g013670.3	*psaN*	Photosystem I reaction centre subunit N
Solyc02g070990.1	*LHCB1*	Chlorophyll A-B binding protein
Solyc02g065400.3	*psbo*	33kDa precursor protein of oxygen-evolving complex
Solyc09g007270.3	*APX2*	L-ascorbate peroxidase 2, cytosolic
Solyc01g067740.3	*SODCC1*	Cu^{2+}/Zn^{2+} Superoxide dismutase 1, cytosolic, chloroplastic
Solyc06g005160.3	*APX1*	cytosolic ascorbate peroxidase 1
Solyc03g034220.3	*rbcS2A*	ribulose bisphosphate carboxylase small chain 2A
Solyc02g085950.3	*RbcS3B*	ribulose bisphosphate carboxylase small chain 3B

Table S2. qRT-PCR primer sequence information

Gene	Forward Primer Sequence（5'-3'）	Reverse Primer Sequence（5'-3'）
Solyc08g013670.3	TGGCTACAAGTGGTGCGAAT	GTCCTTGCCTTCACACTCCA
Solyc02g070990.1	TCTTCCCCTTCTTTTGTTGGG	TCACCAGAGAATGGGCCTAAG
Solyc02g065400.3	CCATTGAAGGAGGTGTTGGC	TGATGTGGGCTCTAGGCAGA
Solyc09g007270.3	TCCGCCCTCTAGTCGAGAAA	CAGCATCTGCAAATCCGAGC
Solyc01g067740.3	TCTCACTGGTCCACAGTCCA	CCAGCATTTCCGGTGCTTTT
Solyc06g005160.3	CATACCTTGGGAAGGGCACA	TTCCCCACTCAAAAGCTCCG
Solyc03g034220.3	TCCTGATTTGTCTGACGAGCAA	CTTGAACCTCAGCCAACACTTG
Solyc02g085950.3	TGCTCAGCGAAATTGAGTACCT	GGTCCAGTATCTGCCATCGTAG
Actin (reference)	CTCTACATACTTGAGAGGTGCC	AGACGAGGAGAAAACATCACAA
